# Role of PKD2 in the endoplasmic reticulum calcium homeostasis

**DOI:** 10.3389/fphys.2022.962571

**Published:** 2022-08-10

**Authors:** Xiong Liu, Jingfeng Tang, Xing-Zhen Chen

**Affiliations:** ^1^ Membrane Protein Disease Research Group, Department of Physiology, Faculty of Medicine and Dentistry, University of Alberta, Edmonton, AB, Canada; ^2^ National “111” Center for Cellular Regulation and Molecular Pharmaceutics, Hubei University of Technology, Wuhan, HB, China

**Keywords:** ADPKD, autosomal dominant polycystic kidney disease, TRPP2 channel, ER stress, PKD2 interacting partner, ER Ca release channel

## Abstract

Autosomal dominant polycystic kidney disease (ADPKD) is caused by mutations in the PKD1 or PKD2 gene which encodes membrane receptor PKD1 and cation channel PKD2, respectively. PKD2, also called transient receptor potential polycystin-2 (TRPP2), is a Ca^2+^-permeable channel located on the membrane of cell surface, primary cilia, and endoplasmic reticulum (ER). Ca^2+^ is closely associated with diverse cellular functions. While ER Ca^2+^ homeostasis depends on different Ca^2+^ receptors, channels and transporters, the role of PKD2 within the ER remains controversial. Whether and how PKD2-mediated ER Ca^2+^ leak relates to ADPKD pathogenesis is not well understood. Here, we reviewed current knowledge about the biophysical and physiological properties of PKD2 and how PKD2 contributes to ER Ca^2+^ homeostasis.

## Introduction

Autosomal dominant polycystic kidney disease (ADPKD) is one of the most common genetic kidney diseases, affecting over 10 million people worldwide, resulting in a huge public health burden and billions of dollars in direct medical costs each year (reviewed in Bergmann et al., 2018). Gene mutations in PKD1 and PKD2 account for approximately 80%–85% and 10%–15% of the ADPKD cases, respectively (reviewed in Al-Bhalal and Akhtar, 2005). PKD1 (also called polycystin-1) encoded by PKD1 is a G-protein like receptor with 11 transmembrane (TM) domains and a large extracellular N-terminus (reviewed in Trudel et al., 2016). PKD2 (also called polycystin-2) is a member of the transient receptor potential (TRP) superfamily of ion channels (thus also called TRP polycystin-2 or TRPP2), which possess six TM (TM1-TM6) domains and play critical roles in a variety of sensations, like pain, touch, temperature, light, and chemicals (reviewed in Vangeel and Voets, 2019). PKD2 is a non-selective cation channel and can form a channel complex with PKD1 that acts as a membrane receptor (reviewed in Hanaoka et al., 2000; Giamarchi et al., 2006; Kim et al., 2016). The PKD2 structure solved by means of cryo electron microscopy (Cryo-EM) shows a homotetrameric organization with a single ion conducting pore, a voltage sensing-like domain (VSLD), and a large extracellular loop between TM1 and TM2 (Shen et al., 2016; Grieben et al., 2017; Wilkes et al., 2017). The complex formed by PKD1 and PKD2 in epithelial primary cilia and plasma membrane through binding between their C-terminal coiled-coil domains was reported to be activated by tubular fluid flow, which allows Ca^2+^ entry thereby initiating downstream Ca^2+^ signaling (Qian et al., 1997; Nauli et al., 2003; reviewed in Ning et al., 2021). However, recent structural studies found that the PKD1/PKD2 complex can still be formed in the absence of their coiled-coil domains (Su et al., 2018), indicating that the two proteins can associate with each other through other domains as well. Altered Ca^2+^ signaling due to dysfunction of PKD1 or PKD2 is thought to initiate cyst formation in ADPKD (reviewed in Mangolini et al., 2016) but the mechanism of pathogenesis remains elusive.

Endogenous PKD2 channel was found to be localized on the plasma membrane and primary cilium membrane of mouse inner medullary collecting duct (IMCD) and Madin-Darby canine kidney (MDCK) cells, while heterologously expressed PKD2 mainly targets to the ER membrane (Koulen et al., 2002; Luo et al., 2003). PKD2 was found to be constitutively active on the plasma membrane of IMCD cells (Luo et al., 2003), while PKD2 heterologously expressed in other mammalian cells such as Chinese hamster ovary (CHO) and human embryonic kidney (HEK) cells, and *Xenopus* oocytes exhibited hardly detectable whole-cell currents, in part because of its relatively low density on the plasma membrane (Arif Pavel et al., 2016; Shen et al., 2016). Perhaps because of this, most functional characterizations of PKD2 were based on single-channel recordings (González-Perrett et al., 2001; Vassilev et al., 2001; Koulen et al., 2002; Cai et al., 2004) rather than measuring whole-cell currents. Interestingly, both single-channel and whole-cilium currents mediated by PKD2 in renal primary cilia were detectable (Kleene and Kleene, 2017; Liu et al., 2018). In 2016, a PKD2 gain-of-function (GOF) mutant F604P was identified and characterized in oocytes in which it displayed large whole-cell cation currents (Arif Pavel et al., 2016). In comparison with the PKD2 structure, we found that mutant F604P is associated with a bent TM5 helix and a π-to α-helix conformational change of TM6, which induces a twisting of the (lower) pore gate residue L677 thereby enlarging the channel pore size (Zheng et al., 2018). Despite of this knowledge on PKD2 gating mechanism, no PKD2-binding agonist has so far been identified. Recently, the extracellular N-terminus of PKD1 was found to activate PKD1/PKD2 complex on the plasma membrane, showing that this complex functions as an outwardly rectifying channel (Ha et al., 2020).

ER as the most important intracellular Ca^2+^ pool plays critical roles in the synthesis, modification, folding, quality control and secretion of proteins (reviewed in Krebs et al., 2015). Conditions disrupting the ER Ca^2+^ homeostasis result in unfolded protein accumulation that constitutes a fundamental threat to all living cells (reviewed in Schröder and Kaufman, 2005). Thus, Ca^2+^ influx and efflux must be precisely controlled because of their important physiological roles in cell adaptation, survival and death (reviewed in Cerella et al., 2010). Intracellular free Ca^2+^ concentration substantially varies from one compartment to another. The Ca^2+^ concentration in the ER lumen can reach 800 μM (Samtleben et al., 2013), which is close to the mM extracellular Ca^2+^ concentrations, while intracellular free Ca^2+^ concentrations are much lower, around 100 nM. This 8,000-fold Ca^2+^ gradient across the ER membrane is equilibrated through Ca^2+^ ATPases and channels on the ER membrane and Ca^2+^-binding proteins in the ER lumen (reviewed in Zhang et al., 2020). Ca^2+^ ATPases such as SERCAs pump Ca^2+^ into ER while Ca^2+^ channels such as ryanodine receptors (RyRs), inositol 1,4,5-trisphosphate receptor (IP3R), PKD2, and translocon complex Sec61 (Lang et al., 2011; Schauble et al., 2012) leak Ca^2+^ out from ER (reviewed in Boyden and Smith, 2018). Ca^2+^-binding proteins such as calsequestrin and calreticulin in the ER lumen serve as Ca^2+^ buffers and stabilize the luminal Ca^2+^ concentration. This review will survey how PKD2 contributes to ER Ca^2+^ leaks by itself or through cross-talks with other ER membrane proteins including IP3R, RYR2, and stromal interaction molecule 1 (STIM1). Relevance of PKD2 to ER stress and ADPKD will also be covered.

## PKD2 membrane localization

**FIGURE 1:**
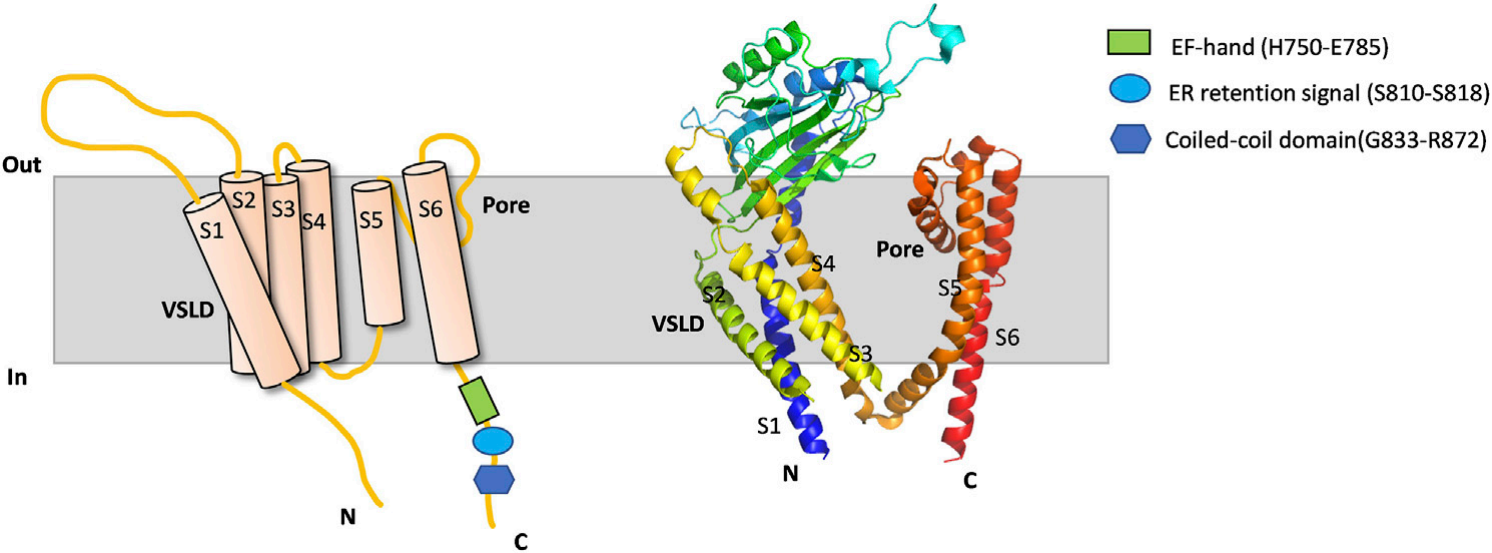
PKD2 membrane topology and Cryo-EM structure. Schematic illustration (left) and cryo-EM structure (PDB: 5T4D) (right) outlining a protomer of PKD2. Membrane, intracellular N- and C-terminus, VSLD, pore domain, TM domain (TM1-TM6), EF-hand, ER retention signal and coiled-coil domain are indicated.

PKD2 is an ion channel that can be localized on different cellular membranes including those of the cell surface, primary cilia and ER (Luo et al., 2003). The membrane localization of PKD2 can vary with different factors, eg due to interaction with cell type-specific chaperones or binding molecules (Liu et al., 2018) or due to indirect factors (Miyakawa et al., 2013). PKD2 contains a C-terminal ER-retention motif (D810-S821) (Figure 1) which can be recognized by phosphofurin acidic cluster sorting protein 1 (PACS-1) and PACS-2. Phosphorylation at S812 by casein kinase 2 (CK2) promotes the PKD2/PACS interaction and ER membrane targeting of PKD2 while PKD2 dephosphorylation at S812 by protein phosphatase represses the PKD2/PACS interaction which facilitates the PKD2 plasma membrane targeting (Köttgen et al., 2005). Consistently, we found that the naturally occurring pathogenic PKD2 truncation mutant R742X missing the ER-retention motif predominantly translocates to the plasma membrane (Chen et al., 2001).

PKD2 was also found to exhibit a dynamic subcellular distribution in a Ca^2+^ dependent manner (Miyakawa et al., 2013). PKD2 targets to the plasma membrane of human and rat primary kidney proximal tubule cells in the presence of an intracellular Ca^2+^ release activator ATP, bradykinin, ionomycin, CPA or thapsigargin. In contrary, intracellular Ca^2+^ release blockers such as BAPTA and 2-aminoethoxydiphenyl borate (2-APB) inhibit its plasma membrane targeting (Miyakawa et al., 2013). Compared to the previously described method of targeting PKD2 to the plasma membrane, localization of PKD2 in primary cilia seems to be regulated by a distinct pathway. In fact, it was found that PKD2 does not traverse the Golgi apparatus on its way to the cilium (Hoffmeister et al., 2011). PKD1 was known to play a chaperone role for PKD2 through forming a channel complex PKD1/PKD2 in the plasma membrane of mammalian cells where the complex was shown to mediate cation currents activatable by Wnt molecules (Kim et al., 2016). However, this finding remains debatable because the agonist role of Wnt molecules has not been reproduced by an independent study (Shen et al., 2016) that showed that, when PKD2 was expressed in either CHO or HEK293T cells with or without PKD1 overexpression, no significant whole-cell current was activated by Wnt proteins.

## PKD2 as Ca^2+^-permeable cation channel

PKD2 was reported to be a Ca^2+^-permeable cation channel in early 2000s (Hanaoka et al., 2000; González-Perrett et al., 2001; Vassilev et al., 2001; Koulen et al., 2002). However, the significance of its Ca^2+^ permeability and whether PKD2 can be called a Ca^2+^ channel has since become controversial (Liu et al., 2018). This study found that the relative Ca^2+^ permeability (P_Ca_/P_Na_ = 0.06) is similar as that of the impermeant N-methyl-d-glucamine (NMDG) (P_NMDG_/P_Na_ = 0.04) determined by patch clamping in primary cilia, suggesting low Ca^2+^ permeability of PKD2 (Liu et al., 2018). On the other hand, the Ca^2+^ permeability of PKD2 was evidenced not only by single-channel electrophysiology using lipid bilayer reconstitution with purified PKD2 or PKD2 in ER-enriched vesicles but also by ^45^Ca^2+^ tracer uptake in *Xenopus* oocytes (González-Perrett et al., 2001; Vassilev et al., 2001; Koulen et al., 2002). Ca^2+^ also exhibits complexed modulatory roles in PKD2: Sub-micromolar intracellular Ca^2+^ activates PKD2 while high [Ca^2+^], like [Mg^2+^], inhibits the PKD2 channel activity. The phosphorylation at S812 by casein kinase II (CK2) was found to play an important role in this bell-shape dependence of the PKD2 function on the cytoplasmic [Ca^2+^] (Cai et al., 2004). In fact, although PKD2 mutant S812A demonstrates similar bell-shape dependence on the cytoplasmic [Ca^2+^], it is 10-fold less sensitive to Ca^2+^ than wide-type (WT) PKD2. It is thus plausible to state that [Ca^2+^] regulates PKD2 trafficking to ER and plasma membranes because the latter was found to be dependent on phosphorylation at S812 (Köttgen et al., 2005).

While PKD2 is permeable to both mono- and divalent cations, using PKD2 GOF mutant F604P, it was shown that the permeation of monovalent cations is inhibited by divalent permeants such as Ca^2+^ and Mg^2+^ (Arif Pavel et al., 2016). Similar inhibition was reported for PKD2 homologue called PKD2-like-1 (PKD2L1, also called TRPP3) (Chen et al., 1999). Indeed, structural analysis of PKD2 in complex with Ca^2+^ and lipids revealed that Ca^2+^ binding at the ion conducting pathway physically blocks the pore, presumably inhibiting ion permeation (Wilkes et al., 2017). Interestingly, functional characterization of a chimera channel made of the PKD2 pore domain plus the PKD2L1 skeleton suggested that PKD2 is more favorable for monovalent cations than Ca^2+^ (Shen et al., 2016). Recently, using radiolabeled ^45^Ca^2+^ uptake in *Xenopus* oocytes as a function readout, we found that PKD1/PKD2 channel complex exhibits higher Ca^2+^ influx than PKD2 expressed alone (Wang et al., 2019) although this is not readily accounted for by structural information (Su et al., 2018). In the absence of PKD1, PKD2 is mainly located on the ER membrane mediating Ca^2+^ leak but its function would be modulated by ER membrane proteins, which will be explored in the next sections.

The PKD2 C-terminal EF-hand motif (H750-E785) was proposed to act as a Ca^2+^ sensor required for channel gating (Figure 1) (Ćelić et al., 2012), which is consistent with the observation that pathogenic mutant L703X missing the EF-hand exhibits no sensitivity to Ca^2+^ (Koulen et al., 2002). However, study using direct patch clamping in primary cilia and knock-in mouse models found that loss of Ca^2+^/EF-hand binding does not significantly alter the channel function or result in cyst formation, and thus proposed that a Ca^2+^-binding site be present outside the EF-hand motif (Vien et al., 2020). Interestingly, we previously found that the EF-hand motif in PKD2L1 is not critical for the Ca^2+^-induced channel activation or the ensuing inactivation in oocytes (Li et al., 2002). Similar results were later reported for PKD2L1 by another group using mammalian cell expression (Decaen et al., 2016).

So far, while the Ca^2+^ permeability of PKD2 has become a consensus how it is regulated on different membranes has yet to be elucidated by further studies.

## PKD2 and IP3R

IP3Rs are ER-localized Ca^2+^-permeable channels activated by IP3, which play an important role in the conversion of external stimuli to intracellular Ca^2+^ signals (Bartok et al., 2019). Several studies showed that PKD2 and IP3R physically and functionally interact with each other to modulate intracellular Ca^2+^ signaling (Li et al., 2005; Sammels et al., 2010a; Sammels et al., 2010b)). In *Xenopus* oocytes and mammalian cells, PKD2 was shown by co-immunoprecipitation to interact with IP3R through the PKD2 C-terminus (Li et al., 2005). Subsequent GST-pull down assays mapped the direct binding domains to the PKD2 C-terminal acidic cluster (810-SEEEDDEDS-818) and to a cluster of cationic residues within the IP3R N-terminal ligand-binding domain (Sammels et al., 2010a). Overexpression of PKD2 significantly prolongs the half-decay time of IP3-induced Ca^2+^ transients, while having no effect on the initial Ca^2+^ release rate, indicating that PKD2 enhances IP3-dependent ER Ca^2+^ release or slows cytosol Ca^2+^ clearance (Li et al., 2015). This regulation of IP3-dependent Ca^2+^ signals by PKD2 should be mediated by their physical interaction because PKD2 pathogenic mutant R742X, which has no physical interaction with IP3R, lost its regulatory ability. Interestingly, overexpressed PKD2 C-terminus also prolongs IP3-induced Ca^2+^ transients, indicating that PKD2-mediated ER Ca^2+^ release is not part of the IP3-dependent Ca^2+^ transients. Reciprocally, the IP3-dependent Ca^2+^ release increases the local cytoplasmic Ca^2+^ concentration thereby activating PKD2, for which the PKD2/IP3R binding is thought to be mandatory (Sammels et al., 2010a). In T lymphocytes, PKD2 was also reported to be localized on ER membrane where it interacts with IP3R to regulate Ca^2+^ release (Magistroni et al., 2019). Consistently, T lymphocytes from ADPKD patients with defective PKD2 exhibits reduced intracellular Ca^2+^ release and elevated cell aggregation and proliferation.

## PKD2 and RYR2

Cardiac RyR2 is a Ca^2+^-induced Ca^2+^ release ion channel located on the sarcoplasmic reticulum (SR) mediating the sarcoplasmic release of stored Ca^2+^ (Otsu et al., 1990). Potential binding between PKD2 and RyR2 was suggested based on the sequence homology of helices TM5 and TM6 between RyR2 and IP3R (Furuichi et al., 1989). In cardiomyocytes, the PKD2 N-terminus was reported to constitutively bind with RyR2 whereas the C-terminus binds with RyR2 in a Ca^2+^-dependent manner (Anyatonwu et al., 2007). This physical binding is essential for PKD2’s blocking effect on the RyR2 channel activity. Compared with control cells, cardiomyocytes with PKD2 knockout (KO) displays higher frequencies of spontaneous Ca^2+^ oscillations and reduced levels of SR luminal Ca^2+^, due to a relief of inhibition of RyR2 by PKD2 (Anyatonwu et al., 2007). Near super-resolution microscopy also confirmed the proximity between PKD2 and RYR2 in cardiomyocytes. It was further showed that inhibition of the SR Ca^2+^ release by PKD2 is through affecting the overall PKA phosphorylation status of RYR2 (Dinello et al., 2020). Consistently, in PKD2 KO zebrafish, the heart also displays impaired intracellular Ca^2+^ cycling (Paavola et al., 2013). Interestingly, the RyR2 protein level is 60% higher in PKD2+/-mice although the mRNA expression is not significantly altered (Kuo et al., 2014), indicating that both the RYR2 expression and function are modulated by PKD2. Altered RyR2 function may trigger a series of molecular events resulting in the development of cardiovascular abnormalities observed in ADPKD patients with defective PKD2 (reviewed in Lemos and Ehrlich, 2018).

## PKD2 and STIM1

STIM1 is a critical Ca^2+^ sensing protein in store-operated Ca^2+^ entry (SOCE) (Zhang et al., 2005). As a single TM protein located on the ER membrane, STIM1 has its N-terminus in the ER lumen acting as a Ca^2+^ sensor and its C-terminus facing the cytosol. Decreased ER Ca^2+^ concentration induces STIM1 polymerization through its C-terminus, which activates a plasma membrane ion channel called Ca^2+^ release-activated Ca^2+^ modulator 1 (Orai1), allowing external Ca^2+^ to enter the cell (Park et al., 2009; Yuan et al., 2009; Zhou et al., 2010). Although it was reported that PKD2 competes with STIM1 for binding with IP3R, no interaction between PKD2 and STIM1 was found using Madin-Darby canine kidney (MDCK) epithelial cells (Santoso et al., 2011). Subsequently, the PKD2/STIM1 association is revealed using over-expressed HEK cells and endogenous vascular smooth muscle cells (VSMCs) (Guo et al., 2020). Thus, whether and how PKD2 and STIM1 interact with each other remains to be determined by further studies. Furthermore, up to now, whether and how PKD2 functionally regulates STIM1, Orai1 or the STIM1/Orai1-mediated SOCE remains an open question. However, due to physical proximity between STIM1 and PKD2, we may envision that PKD2-mediated alteration in the local luminal Ca^2+^ concentration would be sensed by STIM1 thereby affecting its function and thus the function of STIM1/Orai1-mediated SOCE.

## PKD2 and ER stress

As a multifunctional organelle, ER plays critical roles in a variety of cellular processes, including Ca^2+^ homeostasis, lipid and protein synthesis, protein folding, post-translational modification, and regulation of gene expression (reviewed in Bravo et al., 2013). ER stress refers to cellular states in which misfolded or unfolded proteins accumulate in the ER inducing unfolded protein response (UPR) to restore homeostasis or resulting in apoptosis in the presence of prolonged stress (reviewed in Lin et al., 2008). We previously reported that PKD2 is involved in ER-associated degradation (ERAD), which is an essential process for cell homeostasis (Liang et al., 2008a). We found that homocysteine-inducible ER stress protein (Herp), an ER stress marker, promotes PKD2 degradation through Herp/PKD2 association in HeLa and renal cell lines by means of Herp overexpression and knockdown by siRNA or Herp upregulation through ER stress induction. The physical Herp/PKD2 interaction is required because degradation of PKD2 mutants losing Herp association was altered by Herp. Our further study found that PKD2 is able to regulate ER stress, through enhancing the phosphorylation of eukaryotic translation initiation factor eIF2α by one of the three ER stress sensors, pancreatic ER-resident eIF2a kinase (PERK) and that this regulation is through physical association between PKD2 and PERK (Liang et al., 2008b). PKD2 knockdown represses eIF2a phosphorylation but does not alter the PERK kinase activity, indicating that PKD2 facilitates the eIF2a phosphorylation by PERK through enhancing the efficiency of the PERK kinase. Recently, it was also found that PKD2 interacts with transducin beta like 2 (TBL2) to recruit eIF2α to tightly control PERK activation (Zhou et al., 2021). It is well known that increased eIF2α phosphorylation leads to inhibition of proliferation and global translational repression, one of the major characteristics associated with ER stress (reviewed in Park et al., 2019). Up-regulation of eIF2α phosphorylation by PKD2 may represent an important mechanism accounting for over-proliferation seen in ADPKD cells in which PKD2 or PKD1 is defective. It is noted that, while increased eIF2α phosphorylation reduces global protein synthesis it up-regulates the translation of a short list of selected factors such as ATF4, CHOP and Gadd34 that are critical for cells to handle stress conditions through acting on the upstream open reading frames (uORFs) located in the 5′UTR of these factors (Lee et al., 2009; reviewed in Jia et al., 2013). Interestingly, our subsequent study found that P-eIF2α rapidly up-regulates PKD2 protein translation (but not mRNA transcription) by bypassing its inhibitory 5′uORF (Yang et al., 2013), suggesting that PKD2 as a new member in the short list acts as a fundamental molecule for dealing with cellular stress conditions. However, in some pathological states associated with ER stress such as acute kidney injury, non-alcoholic fatty liver disease, and heart and brain tissues under stress conditions, PKD2 mRNA was found to be up-regulated, presumably to prevent from stress-induced cell death (Brill et al., 2020).

## ER PKD2 and ADPKD

As a Ca^2+^ releasing channel in the ER, PKD2 was reported to be regulated by an ER conserved transmembrane protein 33 (TMEM33) and they associate with each other on the ER membrane but not on primary cilia of the renal proximal convoluted tubule (PCT) cells (Arhatte et al., 2019). The PKD2 function is enhanced by TMEM33 as demonstrated by single-channel recordings using lipid bilayer with ER liposomes reconstitution. However, TMEM33 does not exhibit any significant effect on protecting against or rescuing cystic disorders in zebrafish (tail curling and pronephric cyst formation) caused by PKD2 deficiency, suggesting that ER-localized PKD2 may not be relevant to ADPKD. In contrast, the relevance of primary cilium-localized PKD2 to ADPKD has been well known (Nauli et al., 2003; reviewed in Kagan et al., 2017; Kleene and Kleene, 2017). Recently, we found that a membrane protein called TACAN (also called TMEM120A) is colocalized with PKD2 in the primary cilia and that their physical interaction mediates the inhibition of the PKD2 channel function by TACAN (Liu et al., 2021). The channel complex PKD2/TACAN, but not PKD2 expressed alone, is sensitive to pressure, indicating that TACAN may serve as a mechano-sensor. This study further showed that over-expressed TACAN aggravates the tail curling and pronephric cyst formation in larval zebrafish due to PKD2 knockdown by means of CRISPR/Cas9, presumably through repressing the PKD2 channel function. Because of primary cilium and plasma membrane localization of TACAN, this study does not provide supporting evidence for relevance of ER PKD2 to cystic disorders in ADPKD. Further studies are still needed to determine the relevance of ER PKD2 to ADPKD.

## Discussion

PKD2 is a nonselective cation channel with distinct Ca^2+^ permeabilities depending on its subcellular localization and regulation by its binding partners such as TMEM33, IP3R, TACAN, and PKD1. Reversely, PKD2 also acts as a modulator molecule of its partner proteins including IP3R, RYR2, PERK, eIF2α, and STIM1. The channel complex PKD1/PKD2 localized on the plasma membrane and primary cilia of renal epithelial cells is known to be relevant to ADPKD but so far there is no convincing evidence that defective ER PKD2 function can result in any cystic phenotype. The function and regulation of the PKD1/PKD2 channel complex deserve thorough investigations to provide molecular basis for design of drugs that are specific and effective. Similarly, although ER PKD2 acts as a Ca^2+^ release channel either by itself or through partnership with other ER proteins such as IP3R, STIM1, and TMEM33, its physiological and pathological implications have yet to be further determined. Further, based on its crosstalk with factors involved in ER stress and UPR, PKD2 also acts as a fundamental molecule in coping with stress conditions, but leaving an open question as to whether its channel function is essential for fulfilling the role. For this, one would test some loss-of-function point mutants of PKD2 for their ability in dealing with stress conditions. PKD2 is also known to regulate several other cellular functions, such as maintaining cell differentiation and proliferation, induction of autophagy, and developmental signaling in the kidney, heart and reproductive system (Foggensteiner et al., 2000; Ma et al., 2005; Nie and Arend, 2014; Giehl et al., 2017; Peña-Oyarzun et al., 2021). Whether and how the ER PKD2 channel function plays an important role in regulating these cellular functions have to be determined by future studies.
